# High-performance integrated virtual environment (HIVE): a robust infrastructure for next-generation sequence data analysis

**DOI:** 10.1093/database/baw022

**Published:** 2016-03-17

**Authors:** Vahan Simonyan, Konstantin Chumakov, Hayley Dingerdissen, William Faison, Scott Goldweber, Anton Golikov, Naila Gulzar, Konstantinos Karagiannis, Phuc Vinh Nguyen Lam, Thomas Maudru, Olesja Muravitskaja, Ekaterina Osipova, Yang Pan, Alexey Pschenichnov, Alexandre Rostovtsev, Luis Santana-Quintero, Krista Smith, Elaine E. Thompson, Valery Tkachenko, John Torcivia-Rodriguez, Alin Voskanian, Quan Wan, Jing Wang, Tsung-Jung Wu, Carolyn Wilson, Raja Mazumder

**Affiliations:** 1 Center for Biologics Evaluation and Research, US Food and Drug Administration, Silver Spring, MD 20993, USA; 2 Department of Biochemistry and Molecular Biology, George Washington University Medical Center, Washington, DC 20037, USA

## Abstract

The High-performance Integrated Virtual Environment (HIVE) is a distributed storage and compute environment designed primarily to handle next-generation sequencing (NGS) data. This multicomponent cloud infrastructure provides secure web access for authorized users to deposit, retrieve, annotate and compute on NGS data, and to analyse the outcomes using web interface visual environments appropriately built in collaboration with research and regulatory scientists and other end users. Unlike many massively parallel computing environments, HIVE uses a cloud control server which virtualizes services, not processes. It is both very robust and flexible due to the abstraction layer introduced between computational requests and operating system processes. The novel paradigm of moving computations to the data, instead of moving data to computational nodes, has proven to be significantly less taxing for both hardware and network infrastructure.

The honeycomb data model developed for HIVE integrates metadata into an object-oriented model. Its distinction from other object-oriented databases is in the additional implementation of a unified application program interface to search, view and manipulate data of all types. This model simplifies the introduction of new data types, thereby minimizing the need for database restructuring and streamlining the development of new integrated information systems. The honeycomb model employs a highly secure hierarchical access control and permission system, allowing determination of data access privileges in a finely granular manner without flooding the security subsystem with a multiplicity of rules. HIVE infrastructure will allow engineers and scientists to perform NGS analysis in a manner that is both efficient and secure. HIVE is actively supported in public and private domains, and project collaborations are welcomed.

**Database URL**: https://hive.biochemistry.gwu.edu

## Introduction

Many challenges associated with the analysis of extra-large next-generation sequencing (NGS) data result from the size and significance of these datasets. Millions of reads from sequencing runs must be analysed to derive biologically significant meaning from these data, and even more reads are required to discover evolutionary trends through metagenomic level analyses. For example, a comparative analysis of single nucleotide polymorphisms (SNP) profiles for a family of viruses to find determinants of virulence requires parsing of hundreds of millions of reads, tens of genomes and billions of bases, resulting in terabytes of information. This volume is projected to increase to a petabyte scale in the coming years (1–4) with similar trends predicted for most major biological databases (5,6). Application of NGS methods to analyse human genomic or RNA sequences requires additional stringency of methods for deposition, storage and computations of these datasets need to be efficient, to be secure and to have a high level of integrity.

U.S. Food and Drug Administration (FDA) has the responsibility to regulate products that are creating the NGS data, or include NGS data in support of product evaluation. As the underlying technology to create NGS data, as well as the bioinformatics and IT infrastructure required to evaluate NGS data, continue to evolve rapidly, FDA recognizes the need to commit resources to bioinformatics and IT infrastructure. Through a research collaboration with the George Washington University, we report here the development of the hardware and software platform to address these needs. HIVE is the outcome of these activities and represents in-house expertise and the tools FDA needs to evaluate and understand NGS technology. We use HIVE to both support in-house research using and evaluating NGS, and to perform independent analysis as part of our evaluation of NGS data provided to the agency in support of medical product regulatory submissions.

The high-performance integrated virtual environment (HIVE) was created and optimized for the storage and analysis of NGS and other similarly extra-large datasets. HIVE was designed to provide analysis and storage support for NGS data throughout the entirety of its lifespan, by addressing the following needs:

1. Robust retrieval of extra-large data from public and private sources through unstable connection in a concurrent mode: HIVE is compatible with known industry standard sources, is capable of maintaining complicated electronic handshaking protocols, and supporting accession and identifier universe;
2. Distributed storage of extra-large data in a secure environment;
3. High security of proprietary and human derived data while maintaining a collaborative environment where controlled sharing of the data and processes is natively supported;
4. Parallelized computation of data in an efficient manner with high fidelity and traceability;
5. Informative visualization of computational results in a user-friendly interactive manner;
6. Support of custom developed and widely expected external algorithmic tools in an integrated environment;
7. A straightforward pathway for expansion and customization of the library of algorithmic approaches used for data analysis and representation.

Please, see Table 1 for comparison of these and some other features of HIVE platform vs other industry representatives.

**Figure 1.**
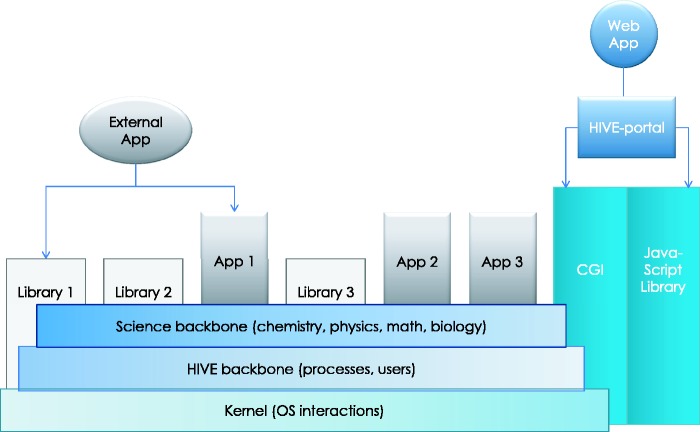
**HIVE backbone**. HIVE core relies on three major components: (1) the kernel layer for low-level interactions with operating system; (2) the native HIVE backbone responsible for distributed storage, security, object model, and computations and (3) a comprehensive science library of functions, data types, and visualizations. A set of task-specific libraries and core applications are available to be used directly or through pipelines for internal and external applications. CGI-based web application layer and custom JavaScript object libraries provide key functionality both for the web portal and for external web applications to interact with HIVE backbone.

Figure 1 shows an overview of the technical organization of HIVE infrastructure. The infrastructure of this environment and the specific solutions HIVE employs to satisfy these requirements will be discussed here in terms of three main tasks: deposition, storage and computation.

**Data deposition:** Challenges surrounding data deposition are largely due to the use of unreliable connections to achieve the reliable transfer of large volumes of data. To address this problem, HIVE uses a multiple attempt procedure with an extensive support system devoted to ensure its success. HIVE can accept and verify files of essentially any size, and ‘fix’ the transfer pathway (if broken), all without direct user involvement. Additionally, the hierarchical security system implemented in HIVE guarantees privacy and security of uploaded files and computations.

**Storage:** In terms of storage solutions, HIVE allows users to save files significantly larger than personal computers typically allow. HIVE stores these files while actively maintaining the linkage between data and metadata, ensuring that the integrity of these files is never compromised.

**Computation:** Although a number of computational methods already exist throughout the NGS industry, HIVE has developed and integrated new and existing tools, optimized for use in a distributed environment, to better facilitate the generation of biologically relevant conclusions from NGS data. HIVE currently provides users analytical capabilities via the HIVE-hexagon aligner (7). (as well as other industry standard aligners), an in house profiler and SNP caller, a sequence manipulation toolbox, a sequence recombination engine and a number of other utilities including a clonal discovery engine, a metagenomics taxonomic profiler (CensuScope (8)), adapted Velvet *de novo* assembler, and others that will be explained in future publications. All tools are developed in or adapted to (in the case of externally developed tools) the cloud cluster environment such that once a computational task is initiated by users, tool-related computations are split into chunks and distributed across hardware, guaranteeing high efficiency and avoiding bottlenecks.

**Figure 2.**
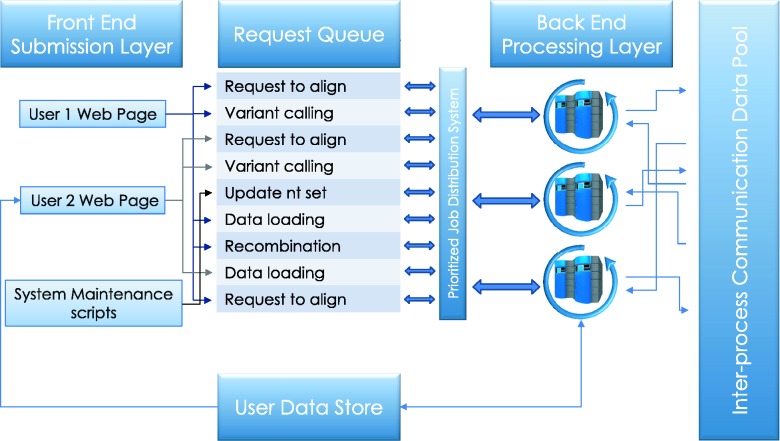
**Service queue**. Job requests submitted by the user or initiated by the system maintenance procedures are queued for processing. Execution priorities are determined by user-initiated load, process type, and, in some cases, user privilege. The cloud control server communicates the availability of tasks to available compute nodes on the back end, which then retrieve the relevant data from the user data store and run executable computations locally. Parallel jobs and sequential processes communicate data and messages through inter-process communication data pool.

Each deployment of a running HIVE engine is capable of queuing (Figure 2) a virtually unlimited number of requests to be run in a resource-managed environment where the amount of memory and disk usage is strictly controlled. As such, HIVE does not overload the computers or networks and the total load is easily configurable by system developers or administrators. The HIVE portal serves as an entry point, in addition to being a tool that redirects the actual storage and computation requests to the nodes which have the lowest load at that particular time. This not only reduces the load on any particular node but also removes the need for extra-large data transfers and long compute queues.

## Materials and methods

### Hardware configuration

HIVE installations are customizable and flexible to meet the needs of many conventional network configurations. HIVE can potentially be run on large enterprise level compute infrastructures as well as on a single computer or set of interconnected computers within private network of organizations. However, the configuration must consider the number of expected total and concurrent users, primary storage space per user, lifespan of computational results, network connectivity, throughput and topology. The system is designed for preferential installation into a Linux environment; however, Mac and Windows installations are also supported.

Using a high performance switch, the HIVE system can connect a virtually unlimited number of computational and storage nodes as a private network. The computational nodes can be standard commercial workstations containing high-performance or retail disk drives. One server acts as both the master job scheduler and the public web interface. The storage nodes are standard commercial networked attached storage (NAS) devices with core processor/s, 2 gigabytes of RAM, and eight 3 terabyte hard disk drives. The NAS devices run a vendor customized version of Linux. Storage is provided to the computational nodes through the network file system protocol using one or many 10 gigabit Ethernet connections. The system allows for ease of expansion and integration of additional nodes, if necessary, in order to increase the computational and storage capacity.

Example implementation of this installment for 50–100 users, 10 concurrent users, that requires no hardware purchases for 1 year (Supplementary Figure S1):

Compute nodes with total of 400 CPU cores with 1600 GB RAM (4 GB RAM, 200 GB local scratch disk space per core)

**Storage server**

- Twenty-four CPU cores, 512 GB RAM. Attached redundant storage: 50 TB permanent, 30 TB scratch, 6 TB staging area
- HIVE domain head node, 16 CPU cores, 24 GB RAM, 2-3 TB local redundant storage with backup. High availability visualization computational node, 10 CPU cores, 256 GB RAM, 2 TB local scratch space
- Web server, 8-16 CPU cores, 32-64 GB RAM, minimum 1 TB storage
- 10 Gbps switch(s) with adequate number of ports to connect all nodes in cluster

**Redundant storage**

- Permanent Storage: minimum recommended 500 GB of disk space per user for NGS data files, reference data (genomes), personal files, and computational results
- Process intercommunication scratch: minimum recommended 300 GB per user, per project
- Data deposition (uploads) staging area: 610 TB depending on connectivity speeds of deposition portals

**Compute nodes**

- Major computational powerhouse nodes: 4 CPU core and 16 GB RAM per user, 1 TB local scratch disk space
- High availability visualization computational nodes: 1 CPU core and 25 GB RAM, 200 GB scratch space per concurrent user
- HIVE domain head nodes: 16 or more CPU cores 24 GB RAM server per redundant sub-domain, 2-3 TB redundant local disk space, depending on anticipated amount of metadata

**Web servers**

- 32 GB RAM, 8 CPU core server with 1 TB local scratch disk space; Minimum two load balanced servers

**Network**

- Appropriate amount of 10 Gbps full duplex switches to accommodate connected hardware
- Firewall (if present) configured to allow connections from cluster to external reference genomic repositories (NCBI, EBI, UniProt, etc.) via HTTP and FTP, ASPERA

**Accounts**

- Single service account across the cluster
- MySQL account
- Email server mailbox access that allow for email storage(i.e. POP3 or IMAP)

### Tools

A number of externally developed applications have been implemented into HIVE and optimized for performance. In addition, several tools have been developed by HIVE and are available through the HIVE interface. Open source tools currently available in HIVE and discussed in this paper include: BLAST (9), Bowtie (10), TopHat (11) and MAFFT (12). Novel tools developed specifically for HIVE and discussed in this paper include: HIVE-hexagon aligner (7), HIVE-heptagon sequence profiler and SNP-caller, HIVE-octagon clustering and classification algorithm, HIVE-seq sequence manipulation toolkit and the recombination discovery engine.

### Public data

Certain HIVE tools use broadly accepted reference datasets as either required or optional inputs for computation. There are two major prerequisite datasets provided by the developers: the National Center for Biotechnology Information (NCBI) nucleotide sequence database, accessed in January 2015 from ftp://ftp.ncbi.nlm.nih.gov/blast/db/, and the NCBI taxonomy dataset, accessed in April 2014 from ftp://ftp.ncbi.nlm.nih.gov/pub/taxonomy/. Other files are shared directly by a HIVE data administrator to minimize redundant use of compute and storage resources. Currently shared public information includes: RefSeq (13) reference genomes of the 12 model organisms, bacterial and viral annotations from NCBI GenBank (14) at ftp://ftp.ncbi.nlm.nih.gov/genomes/ accessed in June 2013, and representative genome sets curated by the Protein Information Resource (15), accessed in March 2013 from ftp://ftp.pir.georgetown.edu/databases/rps/. Please see supplementary information for more details.

## Results and discussion

The unique HIVE architecture described above compared to other widely available tools enhances usability and performance. Below is a detailed description of both the enhanced standard features and entirely novel aspects of HIVE that contribute to its uniqueness as an efficient, massively distributed storage, archival and analysis platform for NGS data.

### How is data deposited into HIVE? Downloader and archiver

The two main components of the data deposition pipeline in HIVE are the downloader and the archiver. The ‘downloader’ allows users to download sequences from the internet or upload sequences from the user’s local environment. Regardless of input, the downloading task is parallelized as much as possible through the creation of concurrent requests (e-utils inquiries or similar) which retrieve the desired data in smaller chunks. Upon retrieval of all pieces, the downloader will validate the completeness of the download and reconstitute the original information. In case of partial or complete failure, the downloader will reinitiate attempts to retrieve missing parts, if any, and upon successful completion, the files will be passed along to the archiver for processing.

**Figure 3.**
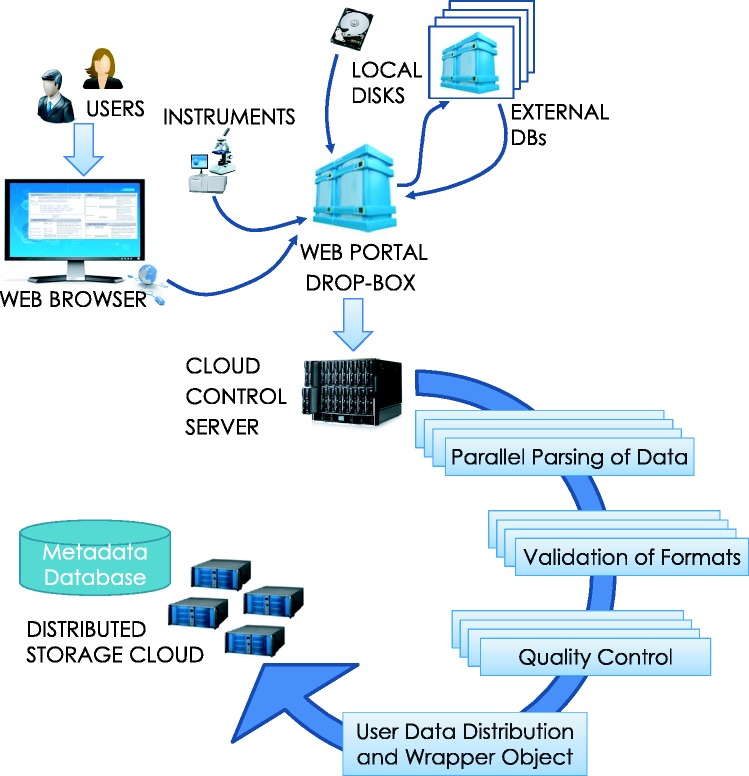
**Data loading process**. Data loading can be initiated by a user upload from local storage or a download request can be submitted to HIVE for concurrent, verifiable retrieval from external sources. Once the data arrives, the HIVE control server initiates parsing, format validation and quality control procedures in parallel before indexing the information. Data are then split and distributed to the storage cloud and encapsulating wrapper objects are created for easy data location and access.

Once files are loaded, the ‘archiver’ accepts parses, indexes, and stores data for future use within the system. HIVE archiver automatically recognizes a large number of generic file formats (txt, csv, doc, jpg, png, etc.), in addition to commonly used file types in the NGS industry: FASTQ, FASTA, FNA/Qual pair, SAM/BAM, VCF, GenBank, properly formatted XML NCBI-bio-project, bio-sample, bio-experiment and bio-run. Single or multiple files can be either loaded in an open native form or inside compressed packages (gz, tar.gz, zip, rar, z2 or bam). Dependent on file type, HIVE applies specific validation, compression and distribution algorithms for the most efficient and minimal-footprint storage. Once retrieved and decompressed, the system executes the parser to identify and analyse the content of each file in order to extract and efficiently store its information. Each file is split, distributed across the storage cluster, and assigned a unique ID that is used internally to recognize and access the specific file-set (Figure 3).

Together these tools manage the storage of any file, regardless of size, in an environment safer than that which conventional personal computers or cloud storage platforms currently provide.

### How is data stored?

Once data has been loaded into HIVE and parsed it is considered to be an ‘object’. Metadata information and field abstraction are implemented by using the HIVE object oriented architecture. Two different kinds of highly optimized database engines are used in HIVE to store structured information: modern relational standard query language (SQL) databases and an internally developed vioDB flat file engine.

To accomplish relational data mapping to object-oriented structures, HIVE implements a flat, ‘honeycomb model’ wherein multiple columns of different tables from an SQL schema are mapped into a single, flat, but highly structured table such that relations between fields are maintained by the honeycomb engine, not by SQL constraints. The honeycomb model minimizes database design and maintenance while providing the capability to implement a single search engine, and visualization and object modification interface to work with data regardless of the internal complexity of the data structure.

Engine allows remembering not only name value pairs of metadata object fields, but also trajectories mapping values onto hierarchical representation of object’s structure. Key/value/path triplets are thus determining the object’s content linking the instance of an object into its type definition which is also maintained inside the same database. The behavior of HIVE objects is as such highly similar to object oriented C/C ++ structure constructs. Type definitions are also objects of a single type and the Honeycomb engine is aware of internal structure. Using that information honeycomb is capable of resolving search queries into multiple joins of SQL in a single transaction.

As an object-oriented data engine, HIVE honeycomb facilitates multi-level single base inheritance and multiple sub-object uses within its class model. This allows developers to define simpler, broader objects and then build progressively more complex subcomponents representing a superset of fields and enriching with more custom field value pairs.

Conventional SQL engines often fail when working with large data sets derived from NGS. To avoid this problem, HIVE implements its own highly indexed, object-oriented database engine and file format called ‘vioDB’ that can manage tera scale metadata. Using relationships between different types of containers and index tables, vioDB allows retrieval of file information in a very efficient way. One-to-many, many-to-one, and many-to-many relations can be supported within heterogeneous types of objects, thus providing direct access to cross-linked information. vioDB can maintain billions of rows of information in a distributed set of files allowing immediate access to any chunk of information using the same honeycomb application programing interface (API).

SQL is of general purpose and is targeted to provide universal functionality for variety of different purposes. That universality frequently comes at a cost of low performance when it comes to indexing and retrieving large tera-scale information. VioDB in contrast is a flat file format, memory mappable container, providing direct access to in-memory content of object values using low level kernel libraries. From API perspective of the application coder the search using vioDB results in memory pointers in page segments, hence no overhead of data transfer, data translation and network transactions. Although not as universal as SQL, vioDB does demonstrate dramatic increase in speed critical for NGS computations using hundreds of parallel processes.

### How are files secured?

Since HIVE is designed for use on unpublished or proprietary data, it is important to have the option of secure storage. HIVE implements a novel security paradigm which is a superset of access control rights subsystems existing in UNIX and Windows systems.

The set of developer-accessible database store procedures and C/C ++ API libraries combined with front-end modules and web application interfaces represent the security gateway controlling and distributing access to the permission subsystem as a whole.

HIVE organizes users into groups located in hierarchically organized projects and groups by assigning single or multiple memberships to its users. Such memberships are audited, verified and approved by group/branch supervisors and by HIVE administrators. HIVE provides a way to dynamically create, move, and invalidate branches upon supervisor request. Additionally, HIVE maintains a separate object-based hierarchy for all entities in the system. Mapping of object privileges to user privileges permits for very fine user access control.

**Figure 4.**
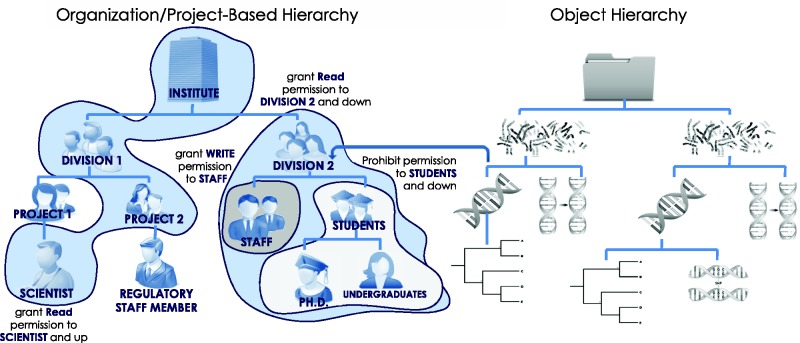
**Hierarchical security model**. Users, groups, files, processes, metadata, visual elements and algorithms are all treated as security-enabled objects. A hierarchical security model allows granting of permissions down the hierarchy, up the hierarchy, or to specific subjects with a minimal number of access control rules. The use of multiple hierarchies to organize users, projects and data objects further controls access rights through the complex mapping between distinct hierarchies.

Objects and object hierarchies shared in HIVE are assigned permissive or restrictive credentials relative to a particular user or branch. Such permission can be non-inherited, inherited down to a group’s members hierarchically or propagated up a chain of supervisors. Thus, a single rule can cover multiple different objects, users and groups hierarchically allowing very granular and targeted permission limitations while maintaining the fewest rules in a collaborative environment (Figure 4). User rules are tracked down in sequence to first ‘allow’, and then ‘deny’ access, avoiding potential unauthorized access leaks.

User-specific viewer masks provide an additional layer of management of access permissions to objects. If a particular user has access to a given object with a mask, only the fields defined in that mask will be accessible for the specified set of permissions. This allows subsets of fields to be visible to a specific subset of user(s) while other users can access different fields within the same object. Refer to supplementary document for a use case and instructions.

The security consideration of data in HIVE is classified based on which of the following categories it belongs to:

***Public data*** are shared, mostly originating from publicly available sources or generated by HIVE authors for the benefit of the HIVE user community. Access to such objects is open to any HIVE user for reading and browsing purposes. A special system user appropriately named ‘biological data handler’ owns the public data and is responsible for maintenance and updates. Examples of such data include but are not limited to public genomes from NCBI, annotations from GenBank or Uniprot, etc.

***Private data*** are that which are uploaded or downloaded by a user from a private repository or local computer, or generated by a user as a result of sequencing and computations. Such data cannot be accessed by anyone but the owner of the data itself, by processes running on his/her behalf, or by system maintenance processes providing backup, encryption or compression functionality.

***Collaborative data*** are intentionally shared by an owner with other users or groups. Such data are accessible only to those who have been assigned appropriate privileges by users who had a right to share the data.

This categorization schema is a policy driven by institutional rules on every particular HIVE deployment host organization.

### How are computations handled in HIVE?

After the deposition and storage of files is complete, users can perform a variety of computational procedures on their data. HIVE computational infrastructure is composed of architecture layers. The biocomputational (BioC) structure is based on cloud technology invented by Dr Vahan Simonyan and implemented together with Dr Raja Mazumder at NCBI/PubChem, FDA/CBER and GWU. BioC works by separating services, requests, data and jobs.

***Services*** are particular implementations of software algorithms. Each service takes predefined inputs and produces known formats of output. Services may have configurations controlling the performance and outcome of the service. A service may be as simple as the parsing of sequence quality files into a workable flat file, or as complex as the alignment of queries on a genome or computation of structural folding of a protein. Services represent an encapsulated package or a layer of encapsulation for packaged, so-called ‘black box’ algorithms that the user of the service does not need to understand in order to efficiently use.

***Requests*** are inquiries to execute service algorithms with specific inputs and configuration parameters. Each inquiry is assigned a unique identification number to track the status of the request and associate the request with the submitter and executor jobs.

***Data*** are generalized arbitrary blobs of information associated with requests and maintained on the BioC server to communicate the users’ inquiry into back-end working processes. Each type of service may have its own predefined structure of data blobs.

***Resources*** are executable images, static data, configuration files and any other form of information needed for proper execution of back-end processes running on powerhouse workstations. The QPride control daemon ensures the workstations dedicated to a particular service always have the resources available on local storage before the execution of process image.

***Jobs*** are processes running under an operating system such as Windows, Linux or Mac. Jobs are launched by the BioC control manager daemon; this design directs processes to grab queued requests for execution and deposit computation results back into the BioC server.

Using this technology, the request to execute an algorithm on a particular data set is communicated back to the highly optimized, parallelized cloud of service-performing jobs. The requests are split for parallelization and queued on the BioC server for execution at the time of node availability.

**Figure 5.**
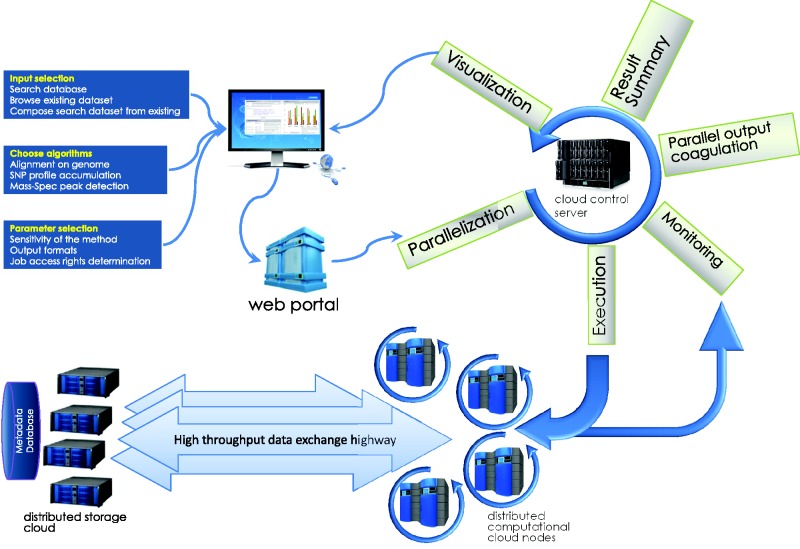
**Computations task information flow**. The submission process, usually a web page CGI process or a client program, submits the initial information from HTML form or web application into the HIVE cloud server queue. The request is then split and submitted to queue for execution. While compute nodes retrieve the data from storage cloud and perform calculations, the front end monitors the status of the jobs and refreshes the status. Once all parts of the computation are finished, data are coagulated and visualizations are prepared and sent to the user’s web page.

Upon submission of HTML-formatted information into the BioC server queue, a unique request identification number is assigned. This identification number allows the system to track the submission and easily retrieve or update associated information. The information flow of the general computation task is shown in Figure 5.

The jobs running on BioC powerhouse computers are designed as daemon processes monitoring the status of the queue for their particular service. Once a request is submitted, a network ping activates the processes to grab the request, retrieve input data, execute the request, upload the results and finally to return to the monitoring state.

For applications to use this infrastructure, API functionality is provided in the form of libraries for C/C ++, shell-executable and Perl scripts. For existing applications already designed without such infrastructure in mind a virtual parallelization layer is used.

Application developers can create new native HIVE programs as well as adopt existing software using HIVE APIs and deploy to existing HIVE instances. HIVE does have large technical documentation describing internals of the system and development guidelines available to its developmental users. However that information is much beyond the scope of this publication and we refrain from putting too much detail and encourage those interested to contact corresponding authors.

### How are existing applications/tools optimized and used in the HIVE environment?

HIVE architecture provides a highly parallel processing environment and distributed data storage not only for custom applications, but for adapted, third-party applications as well. Performance optimization of externally developed tools is achieved by two methods: job-array parallelization and concurrent computations. Job-array parallelization exploits the ability of certain applications or parts of the same applications to be launched in parallel. Results are then combined after completion. This allows HIVE to respond in the same time frame regardless of the total number of users, processes and size of data under computation.

Query data are automatically split into chunks and handled by multiple parallel processes. Generally, each tool outputs alignments in a specific format (e.g. SAM) along with other output files. HIVE merges outputs from the parallel processes and converts the alignments into an internal HIVE format based on the vioDB format (See ‘Viodb file’ definition under ‘How is data deposited into HIVE?’ section above). In this way, the HIVE interface can display alignments produced by internally developed as well as externally developed tools. Other output files from the parallel processes are merged and presented to the user for downloading via the HIVE interface.

The approach described above has been used to integrate several popular alignment tools into HIVE.

***BLAST*** alignment tool by NCBI has been adapted to HIVE (9), optimized to run in a highly parallel fashion, exploiting the advantages of distributed data storage to avoid input/output bottlenecks.

***Bowtie*** is another popular reference-based alignment utility adapted to work within HIVE in a parallel fashion (10). The resulting SAM formatted files are parsed using HIVE’s integrated SAM parsers so users can continue using other alignment-based HIVE virtual services.

***TopHat*** is used for reference-based continuous or discontinuous assemblies (11). It has been adapted to HIVE for RNA-seq analysis. TopHat’s results are also produced in SAM format and HIVE’s SAM parser processes those to a native format and allowing the integration of alignment into pipelines.

### Which analytical tools are currently available in HIVE?

Several tools, both internally and externally developed, are already implemented in HIVE, including DNA-seq aligners, RNA-seq aligners, profilers (pile-up tools), recombination discovery tools, and more. Below is a brief description of some of the most used, currently available HIVE-developed tools.

**Figure 6.**
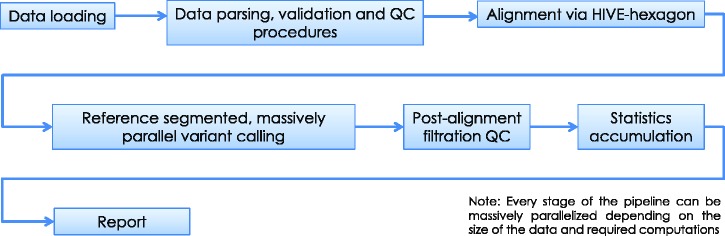
**Alignment pipeline**. The pipeline displayed summarizes the main tasks executed during the sequence alignment process in HIVE. Although we recommend alignment using the native HIVE-hexagon tool, this step can be performed by other industry-standard aligners including Bowtie, BWA and others.

***HIVE-hexagon aligner*** employs new algorithmic approaches which exploit both the nature of extra-large sequencing data and HIVE’s parallel execution architecture to improve upon the speed, sensitivity and accuracy with which alignments are currently performed. Some new and improved utilities which contribute to this enhanced performance include non-redundification and subsequent sorting of sequences using prefix trees, inherent parallelization and an efficient diagonal linearization approach to Smith–Waterman optimization (16). TODO: In Figure 6 once can see the major steps before and after alignment algorithm in a conventional mutation calling pipeline example.

***HIVE-heptagon profiler with genomic-proteomic mapping*** calculates the frequency of individual bases as compared to either a reference genome or consensus sequence. Additionally, the HIVE-heptagon profiler computes quality and noise profiles with appropriate statistics.

To evaluate the impact of identified SNPs on amino acid sequence, HIVE provides graphical representation of the mapping between the genomic and proteomic space. Protein annotation information and metadata are automatically retrieved from the relevant GenBank file upon download, and subsequently parsed by the archiver for quick access during SNP profiling.

HIVE translates the DNA sequence into its corresponding protein sequence based on previously retrieved sequence information and the DNA codon table. This facilitates potential discovery of SNP position mutations which may affect protein structure. When users visualize the profiling output, the system automatically searches for the appropriate annotation file in the user’s space, along with the data regarding the SNP positions. HIVE represents this information in a column graph for SNP positions, side-by-side and in scale to a box plot for protein information. Thus, the distribution of the SNP positions in a DNA sequence and any subsequent effects on related protein sequences can be easily visualized.

***Recombination discovery engine*** allows the user to view variable alignments of the same data to different reference segments, and, through the use of modern graphical functionality, provides clear visualization of any recombination events and their break points.

***HIVE-octagon clustering*** tool performs a comparative analysis of sequence alignment profiling results by hierarchical clustering of SNP frequency data. HIVE’s C ++ API has built-in support for hierarchical clustering of arbitrary biological sequence data, including support for subsequences and sequencing gaps. Pairs of sequences can be compared using a variety of distance functions, such as p-norms, cosine similarity, and Canberra distance. The resulting distance matrix for a set of sequences can be turned into a hierarchical clustering using the neighbor-joining algorithm (17, 18) or the faster but naïve single-linkage and complete-link algorithms. Upon selection of a set of sequence profiling results, produced from alignments against the same reference genome, SNP frequencies at reference positions are interpreted as a new sequence; a hierarchical clustering is calculated for these sequences using algorithms chosen by the user, and the output of results are an interactive phylogram. The phylograms, as well as the raw data used to generate them, can be downloaded for further analysis by external tools.

***HIVE-seq***is a sequence file abstraction layer with sequence manipulation utilities that allow users to merge sequences from different sources into one continuous sequence file object. In addition users can specify the type and amount of noise added to short reads that are generated based on a given genome. The resulting file of short reads derived from the original genome provides a means to measure the quality of different bioinformatics tools. In addition to manipulating reads it also allows users to generate random short reads using a genome sequence, based on user specifications.

As mentioned above, outside tools can be integrated and optimized to capitalize on HIVE’s parallel computational paradigm. All integrations, in a particular HIVE instance, of outside utilities must go through the HIVE development team supporting that instance. This is to ensure the proper integration and security of outside utilities for optimized performance within a HIVE deployment.

### How is the HIVE interface different?

HIVE follows a paradigm similar to Data-Driven Documents (DDD) (19) rather than static HTML pages to address the challenges of visualizations presented by algorithmic outputs from large data sets. DDD is a domain-specific language that enables transformation of the document object model (DOM) driven by data. The separation between content, functionality and DOM is the underlying concept of HIVE’s interface achieved by moving data-binding to the client side. The server sends visualization components that dynamically update the DOM and format the raw data sent to the client. As a result, it is feasible in the HIVE interface to visualize the same content in different ways by simply binding the same data to different visualization modules. In order to maintain and ensure the modularity provided by this paradigm, the communication between client and server is done asynchronously. By incorporating an Ajax (Asynchronous JavaScript and XML) web application model, the HIVE interface allows the user to dynamically exchange data with the server that will in turn update the bounded modules.

HIVE is a cluster of computers that executes a number of heavily parallelized scientific processes. The front end of the cloud (user interface) is a simplified representation of an advanced infrastructure that exists on the back-end. Intermediate layers based on Common Gateway Interface (CGI) and SQL are used in order to achieve the transition of this sophisticated back-end to a user friendly web interface. CGI is used to resolve HTML form data and pass them into the database. Since service requests come in the SQL form, the CGI layer keeps track of every request as it parses it and submits to HIVE. In addition, the database holds the description of the objects within the infrastructure. In that way components like processes, algorithmic or not, are described as objects in the database and can be viewed, edited or inherited by the user through the aforementioned model. As a result, when a user selects to execute a service, an object defined by the database is displayed in the front end; by changing the parameters and submitting the request an object is created in the back-end. As the user waits for the service to be completed, a daemon is responsible for the execution of the service, monitoring the progress, and killing and resubmitting any parts of the parallelized service that have failed. As compute cores complete chunks of the request and report the progress back to the daemon, asynchronous communication between server and front end allows the user to observe the status of the request or even interrupt the process without having to refresh the whole page. (Please see Figure 5 for a schematic of the HIVE backbone.)

### Use case: polio virus strain recombination and mutation profile clustering

HIVE infrastructure allows researchers and their collaborators to perform NGS analysis in a much more efficient and secure manner than is currently possible. (See Table 1 for comparison of features among industry-favorite tools).To fully convey the utility of such an environment, we present here a case study which demonstrates the diverse array of functionalities facilitated by HIVE.

Although poliomyelitis is no longer a major health issue in most regions of the world, new cases are still occurring throughout lower income countries in Africa and the Middle East (20). Of especial concern is the emergence of pathogenic vaccine-derived polioviruses (VDPV) which seem to arise by mutation of attenuated poliovirus strains in regions where vaccine coverage is not high enough (21). Even more disconcerting is the potential for outbreak of infection in regions long thought to be polio-free (22). These concerns have inspired renewed interest in poliovirus transmission and efforts by the U.S. government to review scientific publication and identify research needs.

The complete genomes for 21 previously identified enterovirus strains belonging to the *Picornaviridae* family were downloaded from NCBI GenBank (14) by supplying HIVE’s downloader utility with the appropriate accessions (Supplementary Table S1). HIVE-seq was run on all 21 genomes using the ‘Filters only’ algorithm with default parameter settings to create a single, concatenated genome called ‘Enterovirus_Combined_Genome.fasta’. Ten paired-end deep sequence datasets generated for recombinant VDPV strains isolated in Finland and kindly provided by Dr Merja Roivainen were uploaded from local user space through the HIVE data-loading interface (Supplementary Table S2).

Quality control procedures were automatically performed as reads were loaded and formats were recognized by HIVE. Quality controls consisted of AT/GC content measures, read length histograms, positional distribution of nucleotides and positional distribution of associated quality scores. When viewing the ATGC distribution in the positional QC for samples, it is common to see a bias at the beginning of the chart such that nucleotide content appears to be distributed in a non-random fashion. Primers by definition have non-random content and therefore the appearance of primers within the reads can explain this phenomenon. Primers can be removed using the ‘Primers Filter’ utility from the ‘General’ menu in the HIVE-Portal list of tools and algorithms.

Once confident in the quality of the data, alignments to reference genomes were performed. From the home reads menu, we selected the first pair of reads and clicked the icon to ‘align using HIVE-hexagon.’ Enterovirus_Combined_Genome.fasta, the genome set which we just created, was selected as the reference genome and the alignment was submitted using all default parameter values. Immediately it was seen that this sample contains predominantly sequences similar to Sabin 1, one of three Sabin vaccine strains, with 17 million of almost 27 million reads aligning to this reference. However, there were still significant alignments reported to the related viruses with Sabin 2 having the second largest representation. This suggests a recombination event occurred between strains, but further analysis may be needed to confidently make this claim. (A sample alignment table is included for alignments to ID DQ443002 as a .csv in Supplementary Table S3. Full alignment tables are not included here due to the prohibitive size of such information, but full results can be exported from the web interface if desired.)

Following alignment, we used the HIVE-heptagon profiler utility to accumulate statistics related to coverage, base-calling, and SNP-calling. For the same sample, we saw relatively consistent coverage for the Sabin 1 segment with the exclusion of the 3500–5000 base pair region. To fully elucidate the possibility of recombination at this position, we used the recombination discovery utility. We first created a multiple sequence alignment of the individual references by selecting MAFFT (12) (as implemented within HIVE) from the HIVE-portal menu. The Enterovirus_Combined_Genome.fasta object was selected as the sequence input and the job was launched with default parameters.

**Figure 7.**
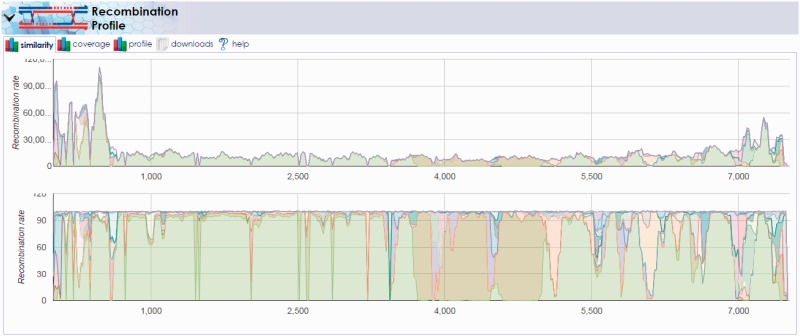
**Recombination discovery between poliovirus Sabin 1 and Sabin 2 vaccine strands**. The output of the recombination discovery utility clearly shows dominant coverage of the Sabin 1 genome (light green) with the exception of the 3500–5000 base pair region. This region, known to contain proteins critical to virus viability, is instead mapping primarily to the Sabin 2 genome (olive green) implying the likelihood of recombination between these two strains in the sample.

Once the multiple-alignment was complete, we launched the ‘Reference Recombination’ tool under the ‘Profiling Tools’ menu on the results page for the Pair_1_Alignment. Here, we selected all reference segments, specified the recently computed MAFFT alignment as the ‘Reference Genome Alignment file’ and submitted the computation with other default inputs. The results demonstrated the recombination amongst the viral strains contained in the reference set, notably the recombination between the Sabin 1 and 2 segments in the 3500–5000 base pair region (Figure 7). The results show that the location of this recombination event is consistent with prior observations of VDPV that often capture non-capsid regions of their genomes from other Sabin strains or other non-polio enteroviruses (23). Repeating this pipeline for all 10 pairs that included VDPVs of all three serotypes revealed a similar pattern of recombination events.

**Figure 8.**
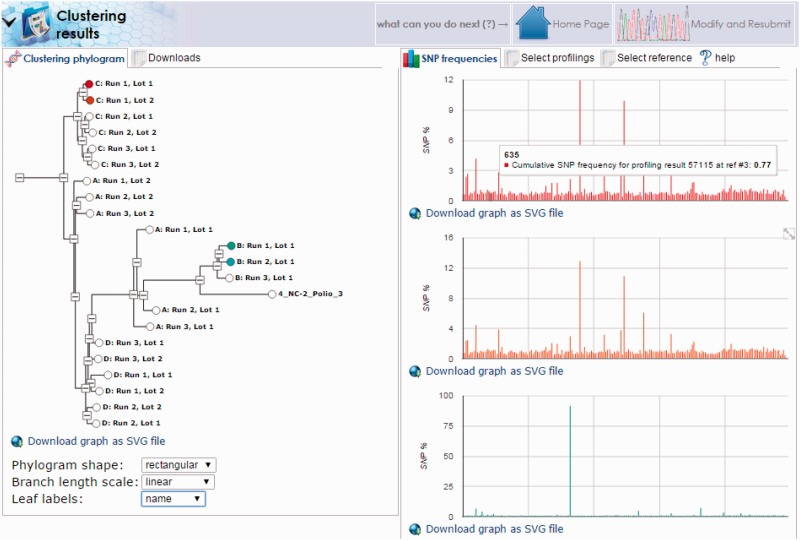
**HIVE-octagon clustering outputs visual comparison of mutation profiles**. Profile clustering of oral polio vaccine samples allows direct comparison of mutation profiles among the samples. Here, we see clusters of each group designated by a letter (A, B, C or D) corresponding to vaccine lots manufactured from four different companies. Clusters are calculated with respect to the occurrence of SNPs called by position. By selecting the nodes in the tree to the left, we can visualize different trends in mutation as a function of SNP frequency by genome position.

Another interesting case involves the stability of viral genomes generated from a single master seed. For this example, data were collected serially from the working seeds of different oral polio vaccine manufacturers generated from the same master seed. Samples were sequenced and aligned as described above to a combined reference containing all three Sabin genomes. Alignments showed a strong similarity among all samples to the Sabin 3 genome with insufficient coverage for the Sabin 1 and 2 genomes. Following alignment, mutation profiles for each sample were generated using the HIVE-heptagon variant caller feature to report mutations with respect to the Sabin 3 genome. All profiles were then entered as input for the HIVE-octagon profile clustering utility which quantifies the difference between mutation profiles as a function of the distance between the mutations. The output is a phylogenetic tree where closer nodes imply greater similarity. The resulting tree (Figure 8) displays not only the grouping of each series (where A, B, C and D correspond to the genomes for vaccine lots of the different manufacturers), but distinctly shows two major clusters. Interestingly, the genome for the group designated by B was determined to be too distant from that of the seed and was therefore never approved for use. This example demonstrates the real-world utility of HIVE workflows in supporting scientific and regulatory NGS research.

### Additional developments and considerations

**Metadata management:** Using convenient and customizable web pages in the HIVE portal, a researcher can create ‘project’, ‘experiment’, ‘run’ and ‘sample’ records in the metadata database to document details of scientific protocols and biological. During this process, identification numbers and names are assigned to metadata records. The scientist can attach those same identifiers to sequencing samples prior to submission to an in-house or out-of-house sequencing center where the resulting NGS data files are produced. When data are available, HIVE pipelines can pick up the files through a secure channel from the data file providers and upload them directly into the HIVE system staging location.

Data are then verified, validated, parsed and appropriate quality control procedures are applied to produce diagrams available for visual inspection by the bioinformatics professional and/or data owner. Once approved and encrypted, the data are split into smaller parts and migrated into the distributed storage system. As with all HIVE data, after the submission is indexed and registered in the metadata archive, the researcher has the opportunity to define security credentials and permission profiles for the data in accordance to institution standards. At this point, data are considered an integrated part of the HIVE storage and are available for searching, downloading or biological curating.

**Visualizations:** Using web-driven visual interfaces, a scientist can search and select one or many data sets for computations. A multiplicity of tools have been created specifically for NGS data analysis, and additional pre-existing, widely used tools specifically optimized for parallelized HIVE infrastructure, can then be used to run computations on the selected datasets. The scope and the number of these tools are not limited and will be updated based on the needs and requests of the scientific community. All data in the system are available for downloading in a variety of industry accepted formats.

**Deployments:** There are currently several distinct deployments of HIVE across two institutions, the U.S. Food and Drug Administration (FDA) and the George Washington University (GWU).

FDA houses two separate HIVE deployments. The first is a cutting-edge research environment that enables rapid development of entirely new NGS analytics algorithms or augmenting function to existing tools. The second is configured in a partially dedicated sub-cluster of a larger high-performance computing cluster at the FDA. Deployment in this environment allows for access to greater compute and storage resources. This deployment is highly secure and allows FDA to store, retrieve, and analyse NGS data provided in support of regulatory submissions.

The public-HIVE located at the Mazumder lab at GWU engages in pilot projects with researchers from several institutes including several other labs from GWU, the Lombardi Cancer Institute at Georgetown University, University of Texas, JCVI and other commercial or academic entities.

HIVE does maintain detail audit log on all compute time and storage capacity per user, per project basis. However all current installations of HIVE are supported by institutions hosting particular hardware and no cost is as such transferred to users.

## Conclusion

The HIVE infrastructure and algorithms provide:

- A high security environment for data deposition, computation, and storage of large NGS datasets, including proprietary and human subject data;
- A mechanism for robust retrieval of NGS data from a variety of sources and the subsequent distributed storage of this data in a highly secure environment;
- An efficient method for data sharing and collaboration;
- A streamlined approach to NGS analysis with fidelity and traceability of all subprocesses;
- Outputs that can be exported for external analysis or viewed internally through a diverse array of high quality scientific visualizations.

The HIVE infrastructure has been developed to address computational challenges associated with the exponentially growing universe of sequence datasets, while maintaining high security of proprietary and private data. HIVE facilitates the robust retrieval of NGS data from a variety of sources and the subsequent distributed storage of this data in a highly secure environment. The HIVE honeycomb data model used to ensure security also enables an efficient method for simple sharing and collaboration through the system. The massively parallel nature of HIVE computations streamlines the process of NGS analysis with fidelity and traceability of all subprocesses. All outputs can be exported for external analysis or viewed internally through a diverse array of high-quality scientific visualizations. HIVE actively seeks and welcomes the opportunity for collaborations to promote a standardized and cooperative NGS community. Please visit https://hive.biochemistry.gwu.edu for more information.

**Table 1.** Key priorities and emphasis of HIVE^a^ compared to other platforms

Features	CLC-Bio	DNAnexus	Galaxy	Geneious	SB Genomics	HIVE
FDA regulatory compliance	−	−	−	−	−	Yes
Data and process security	+	++	−	−	++	+++
Hierarchical sharing^b^	−	−	−	−	−	Yes
Verified data download protocol^c^	−	−	−	−	Yes	Yes
Object traceability/audit^d^	−	Yes	Yes	−	−	Yes
Novel native Next-Gen algorithms^e^	+	−	++	−	−	+++
Login based visibility of tools/databases/interfaces^f^	−	Yes	Yes	−	−	Yes
Distributed storage^g^	−	−	−	−	−	Yes
Parallelization	+	+++	+++	+	+	+++
User App Development	−	Yes	Yes	−	Yes	Yes
User defined metadata^h^	−	−	Yes	−	Yes	Yes
Customizable Visualizations	Client app	Yes	User developed	Client App	Yes	Yes
Mobile apps	−	−	−	−	−	Yes
Amazon EC2	−	Yes	Yes	Planned	−	Yes

a Feature emphasis for other platforms and software are based on information present on website as of September 2015.

b Highly granular sharing mechanism allowing read, write, execute options up and down a hierarchy. Results, processes, tools, databases, data are all objects and can be given/denied access to others.

c Extensive bookkeeping is performed with robust checking to confirm data downloads from public or private sources to ensure all data are downloaded. For big files this is critical as downloads can be interrupted due to connectivity.

d To meet FDA compliance and a need requested by our users in the pharmaceutical industry every process/object is traceable.

e Develop novel algorithms to improve end-to-end NGS analysis.

f Because of hierarchical sharing and almost everything being an object users see on their screen what they have access to.

g Distributed storage makes it possible to efficiently compute and also provides added level of security where if one node is compromised all data are not lost.

h Because users can define metadata it is possible to create databases within minutes which can be integrated into HIVE and used in workflows.

## Funding and Acknowledgements

HIVE is supported by funding from Medical Countermeasures Initiative and in part by R.M. research funds. We would like to acknowledge the following people for providing feedback and HIVE support over the years: Robert Foreman, Garrett Fields, Eric Donaldson, Fan Yu, Hu Yu, Brian Fitzgerald, Darren Jansen, Mark Walderhaug, Christopher Kiem, and other colleagues who have tirelessly supported HIVE efforts.

## Funding

This research was supported in part by the Food and Drug Administration Medical Countermeasures Initiative. The funders had no role in study design, data collection and analysis, decision to publish, or preparation of the manuscript.

## Supplementary data

Supplementary data are available at *Database* Online. 

*Conflict of interest*. None declared.

Supplementary Data
