# Supplementary material for: High-performance integrated virtual environment (HIVE): a robust infrastructure for next-generation sequence data analysis
Source: Database (Oxford). 2016 Mar 17;2016:baw022. doi: 10.1093/database/baw022 (PMC4795927; doi:10.1093/database/baw022)
Supplement: Supplementary Data [file supp_2016_baw022_index.html]

Supplementary Data 

# High-performance integrated virtual environment (HIVE): a robust infrastructure for next-generation sequence data analysis

## Supplementary Data

files

- Supplementary Data - zip file
