# Supplementary material for: High-performance integrated virtual environment (HIVE): a robust infrastructure for next-generation sequence data analysis
Source: Database (Oxford). 2016 Mar 17;2016:baw022. doi: 10.1093/database/baw022 (PMC4795927; doi:10.1093/database/baw022)
Supplement: Supplementary Data [file supp_baw022_suppl_data.zip › HIVE_Infrastructure_Reviewer_read-me.docx]

To access the tools and data referenced in the manuscript, please go to the following URL:

<http://hive.biochemistry.gwu.edu/review/HIVE_>Infrastructure

The above URL will log you in and take you directly to the HIVE_Infrastructure folder in the file directory for the **hive.reviewer** user. This folder contains all data referenced in the manuscript, named as specified by the manuscript and by supplemental information below. In addition to datasets, you will also have access to the computational (alignment, profile, clustering) results discussed.

If you have any issues with the link due to firewalls, etc., please contact one of the corresponding authors and he will provide you with temporary authentication credentials.

Contact:

Vahan Simonyan, Ph.D.

240-888-3570

[vahan.simonyan@fda.hhs.gov](mailto:vahan.simonyan@fda.hhs.gov)

Raja Mazumder, Ph.D.

202-994-5004

[mazumder@gwu.edu](mailto:mazumder@gwu.edu)

TO BROWSE FILES

When logged in through the URL, you should see the HIVE_Infrastructure folder highlighted (purple) in the left-hand home directory menu under the HIVE Space directory. If the folder is not highlighted it is not properly selected. You can select a folder by clicking on its name. Once selected, the files listed on the right should be all those files relevant to this manuscript. You may view all objects in one list (All), only genomes (Genomes), only read datasets (Reads), or only files (Files). Computations may also be viewed by clicking the Computations tab at the right of the directory toolbar. Here you will have access to all alignment, profile and profile clustering results referenced in the paper.

SUMMARY OF AVAILABLE FILES (See Supplemental tables for additional details)

23 Genomes

64 Reads

70 Computations (33 HIVE-hexagon, 33 HIVE-heptagon, 1 HIVE-octagon, 1 HIVE-Seq, 1 MAFFT, 1 Recombination)

3 Files (1 read-me .docx, 1 supplemental spreadsheet .xlsx, viodb.zip)

SUMMARY OF GLOBALLY AVAILABLE FILES

Reference genomes for model organisms were directly accessed on the following dates using the bioproject IDs listed below: Mus musculus, ID 169, December 2013; Rattus norvegicus, ID 12455, December 2013; Gallus gallus, ID 10808, December 2013; Danio rerio, ID 13922, December 2013; Drosophila melanogaster, ID 164, March 2013; Schizosaccharomyces pombe, ID 127, March 2013; Saccharomyces cerevisiae, ID 128, March 2013; Homo sapiens, ID 168, March 2013; Escherichia coli str. K-12 substr. MG1655, ID 57779, March 2013; Dictyostelium discoideum, ID 13925, March 2013; Caenorhabditis elegans, ID 158, March 2013; Arabidopsis thaliana, ID 116, March 2013. All above mentioned datasets are updated periodically based on NCBI release calendar.

TO COMPUTE ALIGNMENT

If you would like to replicate a computation, click on HIVE-Portal to open the HIVE-Portal menu. Next click Alignment and then choose the appropriate aligner. HIVE-hexagon runs for this publication were performed using the corresponding Viral mode.

HEXAGON INSTRUCTIONS

1. Specify a name for your alignment.
2. Select your data set (reads) from the Sequence Read dropdown menu.

Note: Reads and genomes are organized the same as in user home directory. When selection window pops up, expand HIVE Space by clicking black arrow, then click HIVE_Infrastructure. Close selection window by clicking red X in top right corner.

1. Select your reference (genome) from the Reference Genome dropdown menu.
2. Minimum match length was set to 75 for all runs discussed in the paper.
3. Select Random vote between equally best alternative matches for Matches to Keep.
4. Leave percent mismatches allowed as default (15).
5. Once all parameters are specified, ALIGN button will appear. Click. Results will populate when complete.

HEPTAGON INSTRUCTIONS

1. From the hexagon results page select “Sequence Profiling” from the Profiling Tools menu
2. Leave all parameters at default values and click the “Analyse All References” button.
3. Click “OK” to continue.

OCTAGON INSTRUCTIONS

1. From the heptagon results click “Hierarchical Clustering” to the right of “what can you do next?”
2. Select your desired reference genome (in our case, Sabin123.fasta) from the dropdown menu
3. Select your desired reference gene (in our case, Sabin 3) from the dropdown menu
4. Under the dropdown menu for HIVE-Heptagon profiling results to compare, select all profiles for the sample data (23)
5. Click the Compute button.
