# Supplementary material for: High-performance integrated virtual environment (HIVE): a robust infrastructure for next-generation sequence data analysis
Source: Database (Oxford). 2016 Mar 17;2016:baw022. doi: 10.1093/database/baw022 (PMC4795927; doi:10.1093/database/baw022)
Supplement: Supplementary Data [file supp_baw022_suppl_data.zip › FigureS1.pptx]

## Slide 1
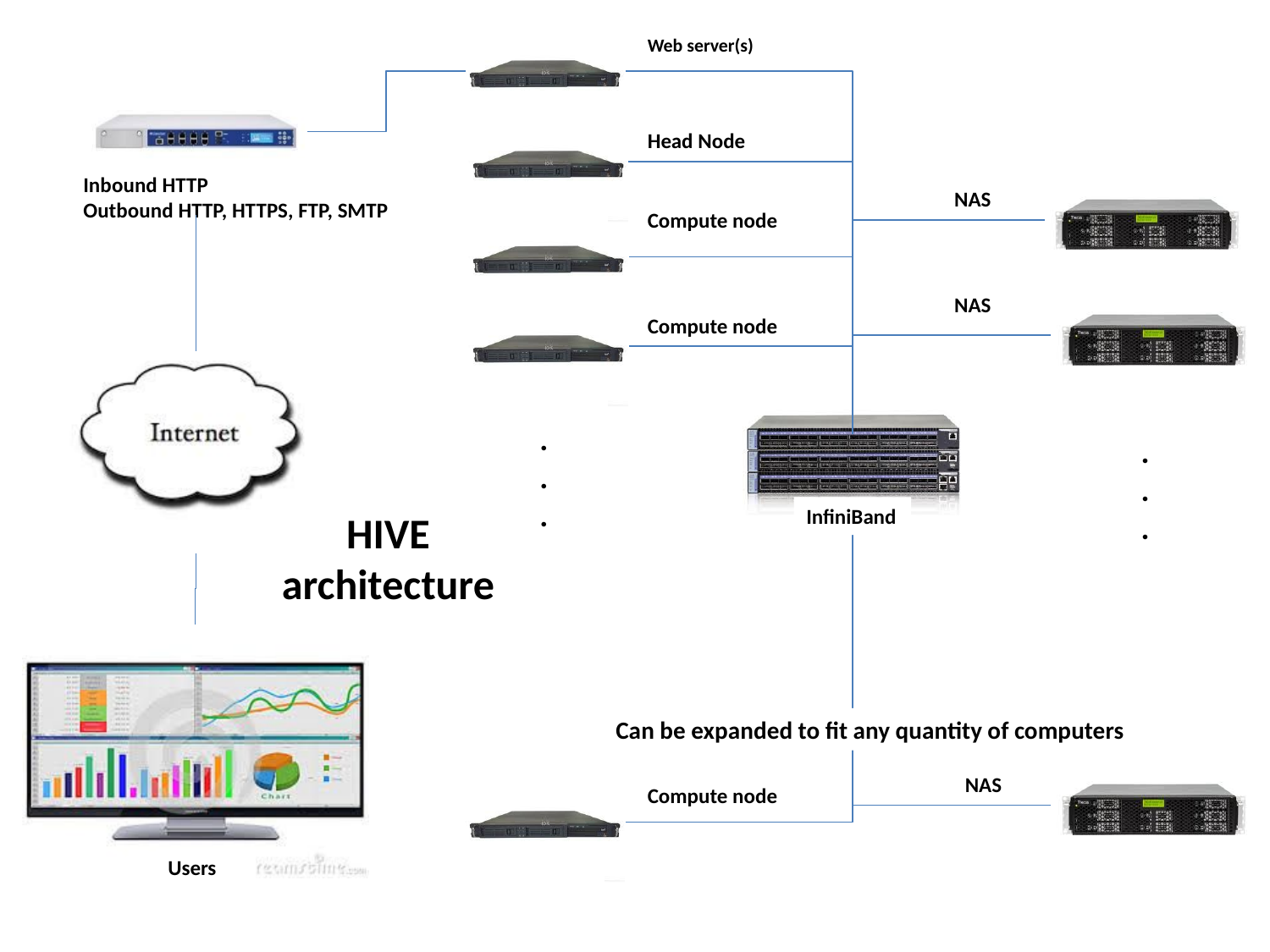

Web server(s)
Head Node
Inbound HTTP
Outbound HTTP, HTTPS, FTP, SMTP
NAS
Compute node
NAS
Compute node
.
.
.
.
.
.
InfiniBand
HIVE
architecture
Can be expanded to fit any quantity of computers
NAS
Compute node
Users

## Slide 2
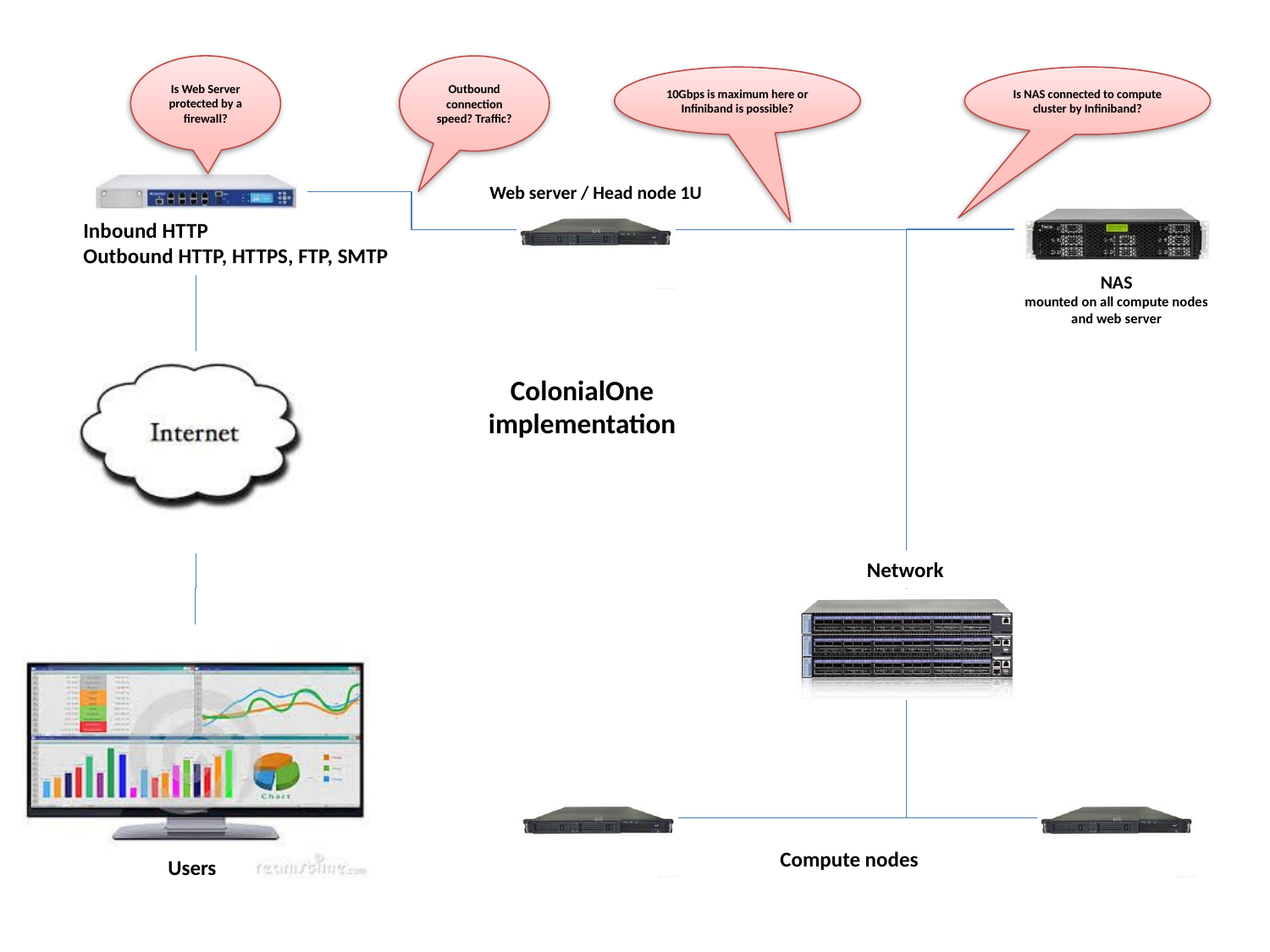

Is Web Server protected by a firewall?
Outbound connection speed? Traffic?
10Gbps is maximum here or Infiniband is possible?
Is NAS connected to compute cluster by Infiniband?
Web server / Head node 1U
Inbound HTTP
Outbound HTTP, HTTPS, FTP, SMTP
NAS
mounted on all compute nodes and web server
ColonialOne implementation
Network
Compute nodes
Users
